# Is early childhood exposure a key predictor of adulthood problematic gaming?

**DOI:** 10.1371/journal.pone.0348901

**Published:** 2026-05-26

**Authors:** Shannon A. H. Compton, Paul F. Tremblay, Elizabeth A. Osuch, Derek G. V. Mitchell

**Affiliations:** 1 Graduate Program in Neuroscience, Schulich School of Medicine and Dentistry, University of Western Ontario, London, Ontario, Canada; 2 Brain and Mind Institute, University of Western Ontario, London, Ontario, Canada; 3 Department of Psychology, Faculty of Social Science, University of Western Ontario, London, Ontario, Canada; 4 Department of Psychiatry, Schulich School of Medicine and Dentistry, University of Western Ontario, London, Ontario, Canada; 5 First Episode Mood and Anxiety Program, London Health Sciences Centre and Lawson Health Research Institute, London, Ontario, Canada; 6 Department of Anatomy and Cell Biology, Schulich School of Medicine and Dentistry, University of Western Ontario, London, Ontario, Canada; City University of New York, UNITED STATES OF AMERICA

## Abstract

Internet Gaming Disorder (IGD) is a proposed condition wherein a preoccupation with video games causes distress and functional impairment. However, the extent to which IGD resembles substance use disorders is controversial. In substance-related disorders, the timing of first exposure correlates with adult symptom severity; however, it is unclear whether IGD symptoms share a similar pattern. The present study used growth mixture modelling to identify how the onset of frequent gaming during early life relates to problematic gaming in adults. A four-class model that differentially predicted current IGD symptoms was identified. The ‘Consistently High Group’ (high levels of gaming during childhood and adolescence) displayed higher IGD symptoms than the ‘Low Escalating Group” (low gaming during these stages) and ‘Rapidly Escalating Group’ (low gaming during preschool followed by high gaming). However, the ‘Consistently High Group’ did not show greater IGD symptoms than the ‘Moderate Group,’ which was the only other group with substantial preschool gaming. Regression analyses confirmed that gaming during preschool and high school predicted current IGD symptoms, with preschool gaming being the strongest predictor. This study highlights additional parallels between substance-related disorders and IGD, specifically identifying an early age of onset as a predictor of adult symptom severity. These results may inform caregivers and paediatric guidelines concerning when and how video game play is introduced and motivate further research clarifying how problematic gaming may emerge through interactions between developmental exposure, mental health, environmental factors, and individual traits.

## Introduction

Non-substance related disorders (NSRDs), sometimes called behavioural addictions, are repeated behaviours that persist despite the knowledge that these behaviours have negative consequences [1]. The most recent edition of the *Diagnostic and Statistical Manual of Mental Disorders* (DSM-V-TR) includes only gambling disorder under the new NSRD classification. However, Internet Gaming Disorder (IGD), a preoccupation with video games to the point of distress and functional impairment, has been included in the DSM-V under “Conditions for Further Study”, a section for proposed conditions that require further research before being included as a formal diagnosis [2,3]. Like gambling and substance-related disorders, there is evidence that IGD features all six components outlined in the components model of addiction [4], including salience, mood modification, tolerance, withdrawal, conflict, and relapse [5,6], though withdrawal symptoms may be less severe for gaming [7]. However, the APA has highlighted the need for more research into the clinical course and risk factors of IGD before its inclusion in the next edition of the DSM [2,3].

Despite some commonalities with substance use disorders, the idea of classifying NSRDs as a mental disorder is controversial. On one hand, there are concerns that such inclusions may lead to pathologizing common behaviours because of a lack of consensus on the definition or theoretical framework for NSRDs [8,9]. It is also argued that problematic levels of gaming are an expression of an underlying mental health issue (e.g., depression), rather than a disorder in itself [10]. However, others note that NSRDs also feature the core elements of addiction including psychological distress [11,12], performance disruptions at school or work [7,13], and social conflict [14,15]. Furthermore, NSRDs may share similar neurobiological risk factors and features as substance related disorders including abnormalities in reward processing, emotion regulation, and decision making [16]. These qualitative similarities in presentation between IGD, gambling disorder, and substance-related disorders raise questions about whether they also show overlap in terms of other outcomes and risk factors.

One potential parallel between substance-related disorders and IGD yet to be explored is the developmental risk factor of age of exposure to the target of addiction. Early exposure to drugs of abuse has been identified as the best predictor of adult dependence [17] and has been associated with worse outcomes in later life [i.e., more severe dependence; 18,19]. Furthermore, despite high rates of drug experimentation among adolescents, only a fraction develop substance use disorders, typically those with the earliest age of exposure [20,21]. Similarly, early onset of gambling behaviours (before age 14) has been consistently identified as a predictor of later problematic gambling [22], and has been associated with increased gambling severity and psychiatric treatment [23]. Examining age of onset may be particularly salient for IGD as, generally, people start playing video games at a much younger age than when they first drink alcohol (13 years; [24]) or use hard drugs (15 years; [25]). Longitudinal research relating early gaming behaviours to later IGD is extremely limited; however, one 6-year study found that those who had pathological levels of gaming at age 20 had shown stable symptoms of pathological gaming from ages 14–18 [26].

To our knowledge no study has yet characterized video game engagement throughout childhood and adolescence to determine how the onset of frequent gaming is associated with later life IGD symptoms. Although this is unsurprising given the logistical challenges and expense of longitudinal studies, there is also a lack of retrospective studies that cover the lifespan. This may be due in part to the unavailability of a measure that establishes a complete record of video game engagement throughout early development to the present. There are questionnaires that assess current [27] and some past video game usage [28]; however, such questionnaires do not measure video game usage before grade 7 (approximately 12 years old). This is important because, although data is sparse, evidence suggests that gaming exposure is frequently initiated by age 5 [29]. Strikingly, although paediatric medical guidelines state that gaming is not recommended before age 2 and should be limited to a maximum duration of one hour a day between ages 2 and 5 [30], there is a lack of empirical data related to the safety of this recommendation where it concerns gaming. To help address this knowledge gap, we developed the Lifetime Video Game Usage Scale (LVUS), a comprehensive assessment of video game engagement from preschool to the present. Recognizing parallels with the substance use literature, we first identified similar retrospective measures of substance use and addiction [NIMH Life Chart: [31], Lifetime Substance Use Recall Scale: [32]] and incorporated language similar to that used in existing video game questionnaires [28]. To support accurate recall, the LVUS asks participants to estimate how many days per month they played video games, rather than how many hours they spent playing. Research suggests that recalling frequency requires less attentional resources than duration [33–35], which may lead to more accurate reporting. With the LVUS, we examine another potential similarity between IGD and substance-related disorders by establishing whether an early onset of frequent gaming predicts adult IGD symptoms.

As with substance-related disorders, it will be important to identify contextual risk factors that predispose individuals to later NSRDs. One potential risk factor is the social context in which the addictive behaviour takes place. Initially, it was thought that problematic gaming behaviours were predominately an issue for online gamers, which prompted the use of the term ‘Internet Gaming Disorder’ (APA, 2013, 2022). However, the suitability of this term has been questioned, since online and offline gaming are weighted equally in the diagnostic criteria [36,37]. One difference between these two gaming formats is that there is often a considerable social aspect to online gaming, and social motivations have been linked to problematic gaming behaviours [38]. Social motivations are also common in the substance-related disorder literature [39]. Yet, paradoxically, addiction is more closely related to solitary substance use [40,41]. It remains unclear whether the social context of gaming influences the risk for and severity of problematic gaming. Delineating the relationship between IGD symptoms and gaming in online or social contexts could provide some clarity as to which criteria would be significant for diagnostic classification.

### The present study

The present study applied growth mixture modelling (GMM) to determine whether differences in the onset of frequent gaming during childhood and adolescence, as measured by the LVUS, predicts IGD symptom severity in adulthood. Given evidence that considerable variability in longitudinal gaming trajectories exists [26], the ability of GMM to identify a number of unobserved subpopulations within a larger group that each follow distinct developmental trajectories [42] makes it well-suited to address the objectives of the current study. Exploratory analyses were conducted to help identify contextual risk factors in present day gaming, including online versus offline gaming and solitary versus social gaming. Our central hypothesis was that frequent gaming during early development is a risk factor for later problematic gaming behaviours. Specifically, we predicted that high video game engagement during the earliest life stages would be the strongest predictors of current problematic gaming symptoms. Additionally, we predicted that online gaming and solitary gaming would be associated with more severe IGD symptoms than offline gaming or gaming with others.

## Methods

### Participants

This study was approved by the Office of Human Research Ethics at the University of Western Ontario under protocol number 119623; data collection took place between January 2022 and February 2023. Eligible participants resided in Canada or the United States, were between 18 and 40 years of age, and reported no current mental health or serious physical health conditions. Participants were recruited through the online participant sourcing platform CloudResearch, which improves data quality by using an approved participants list which excludes participants that fail background security checks, use VPNs, or use insecure IP addresses. Participants provided informed consent by digitally signing (by button click) the Letter of Information on Qualtrics before completing the questionnaires in the first questionnaire session. Consent was verified by the research team. Our sample size was chosen based on recommendations concerning the accurate identification of latent classes through common information criterion using GMM [43]. As such, 500 healthy adults (M_age_ = 32.2 years, SD = 4.83, 60.6% male) completed the screening questionnaire and LVUS, and were invited to complete a longer questionnaire session, which was completed by 437 participants (M_age_ = 32.2 years, SD = 4.70, 61.6% male). Further, to evaluate the robustness and generalizability of the GMM solution, the chosen model was also applied to an independent validation sample (N = 202).

For the regression analyses, sample size was calculated based on the medium effect sizes (r ranging from 0.2–0.3) of our predictors of interest found in previous research relating gaming history [44] and current gaming behaviours [36] to IGD symptoms. Our target sample size selected based on the needs of GMM is far larger than the recommendations for a linear regression analysis with four predictors, wherein a sample of 84 is required to achieve a power over 0.80 [45].

### Procedure

Potential participants responded to a post on CloudResearch describing a research study investigating “digital media preferences and cognitive and social styles” and completed an online screening questionnaire administered through Qualtrics. Eligible participants were invited to participate in the context of a larger study about the impact of gaming frequency. While the larger project consisted of two virtual sessions (one devoted to the current project and another session related to a study examining empathy task performance), this project only includes data collected during the initial questionnaire visit. The results of the empathy study visit are distinct and will be presented elsewhere. Participants completed this session remotely on their own devices with the session taking approximately one hour to complete.

### Questionnaires

#### Internet gaming disorder assessment.

To measure problematic or addictive gaming behaviours, participants completed the Internet Gaming Disorder Scale – Short Form (IGDS9; Pontes & Griffiths, 2015). The IGDS9 is a 9-item questionnaire, where each item measures one of the nine diagnostic criteria of IGD in the DSM-V-TR including gaming preoccupation, tolerance, and withdrawal (APA, 2013, 2022). The IGDS9 quantifies the severity of IGD symptoms by using a 5-point Likert scale to assess gaming related behaviours over the past 12 months (1 = Never to 5 = Very Often). Prior work shows that the IGDS9 has a high internal consistency (Cronbach’s alpha = 0.87; Pontes & Griffiths, 2015) and has been shown to be reliable across samples (Pearson separation reliability = 0.86; Poon et al., 2021).

#### Video game history assessment.

The LVUS consists of 9-items asking individuals about their video game usage over the past 12 months and at previous life stages. For recent gaming, participants were asked “On average, how many hours per week did you play video games over the past 12 months?” This was then broken down to a) last 3 months, b) 4–6 months ago, c) 7–9 months ago, and d) 10–12 months ago. As the upper age range of our sample was 40, participants were asked about their gaming history for the following life stages: preschool (ages 0–5), early elementary (ages 6–9), late elementary (ages 10–13), high school (ages 14–18), young adulthood (ages 19–23), early adulthood (ages 24–29), early 30s (ages 30–34), and late 30s (ages 35–39). For each life stage, the questionnaire asked participants “During [life stage], about how many days per month did you play video games?” Additionally, participants were asked “During [life stage], how many hours did you play video games, on average, on the days you did play video games?” For the current study, we focus only on the first four life stages, which asked participants about their video game use during preschool, early elementary, late elementary, and high school. Because the age range of our sample was 18–40, everyone in our sample had completed these life stages. To establish the reliability of the measure, 120 participants (M_age_ = 31.9 years, SD = 4.74, 69.2% male) were randomly selected to complete the LVUS a second time, with an average interval of 68 days (range 60–90 days) between test and retest sessions. Both Spearman and intraclass correlation coefficients (ICC) were used to establish the measure’s reliability. Reliability correlations of .70 are considered acceptable reliability [47] and ICCs were computed for absolute agreement, which are considered “moderate” between .50 and .75, and “good” between .75 and .90 [48]. The Spearman correlations and ICCs for the hours per day questions approached but did not meet acceptable reliability (i.e., < .70; Calamia et al., 2013). Consequently, analyses regarding time spent gaming at different life stages were conducted using the data from the days per month questions (Table 1).

**Table 1:** Test-retest data for the first version of the Lifetime Videogame Usage Scale. A subsection of participants completed the questionnaire a second time (average interval = 68 days).

Life Stage	*r_s_*	ICC
Preschool (ages 0–5)	.83***	.70***
Early Elementary (ages 6–9)	.82***	.75***
Late Elementary (ages 10–13)	.72***	.68***
High School (ages 14–18)	.71***	.69***
Total (Sum)	.85***	.78***

n = 120; * p <.05; ** p <.01; *** p <.001.

To characterize participants’ recent gaming behaviours, participants indicated how many hours they spent gaming each week in recent months and the internet and social contexts of their gaming. To assess internet gaming, they were asked: “Of the games you played, what percentage of the time did you spend 1) Playing games offline and 2) Playing games online”. To assess social gaming, they were asked: “Of the games you played, what percentage of time did you spend 1) Playing alone, 2) Playing with people you don’t know, 3) Playing with people you know only ONLINE (i.e., have not met in person), 4) Playing with people you know IN PERSON?”

### Data analysis

#### GMM.

GMM was used to identify latent subgroups with distinct trajectories of video game play across developmental life stages. Standard growth models assume a single trajectory for all participants, but prior research suggests variability in longitudinal gaming trajectories [26]. GMM captures this heterogeneity by estimating multiple latent classes, each with its own intercept, slope, and quadratic term.

A single-trajectory model of the first four life stages (i.e., preschool, early elementary, late elementary, high school) was conducted using MPlus 8.7 [49] to determine whether the overall sample trajectory of video game play over time displayed an overall linear or quadratic trajectory. The best fitting models are those which have a significant χ^2^ difference, a smaller Bayesian Information Criteria, a comparative fit index (CFI) and Tucker-Lewis index (TLI) >.90, a root mean squared error approximation (RMSEA) <.09, and a standardized root mean square residual (RMSR) <.08 [50,51].

Growth mixture modelling was then conducted to identify any significant variation in individual trajectories that could be explained by two or more unique trajectories of video game play frequency across the four life stages. Participants are assigned to a class based on their most likely class membership (i.e., the class with the prototypical trajectory they match most closely). Using GMM, a one-, two-, three-, four-, five-, and six-class model were tested to determine the optimal number of classes. The best fitting model was selected by referencing fit indices including the Akaike Information Criteria (AIC), the Bayesian Information Criteria (BIC), the sample-size adjusted BIC (aBIC), entropy, the Vuong-Lo-Mendell-Rubin likelihood ratio test (VMR LRT), the Lo-Mendell-Rubin likelihood ratio test (LMR LRT), and the bootstrap likelihood test (BLRT). Models with lower information criterion (IC) indices, entropy values closer to 1.0, and significant likelihood ratio tests (<.05) indicate better model fit [42]. A Bolck-Croon-Hagenaars (BCH) procedure was also included to test whether classes differed based on IGD scores. The BCH procedure is a 3-step method which relates class membership to a distal outcome (IGD scores in this case) after the classes have been identified as not to affect class membership while correcting for possible classification error [52].

#### Statistical testing.

The remaining analyses were conducted using IBM SPSS Statistics 29 [53].

A series of linear regressions were performed to determine the relationship between IGD scores and: 1) video game engagement during the first four life stages, 2) offline versus online gaming, and 3) different social gaming behaviours. For the first regression, predictors were days spent gaming during the first four life stages (preschool, early elementary, late elementary, high school). For the remaining analyses, new variables were created by multiplying the amount of hours participants reported playing in recent months by the percentage of time they reported spending on each activity (e.g., 10 hours gaming/week x 50% of the time spent gaming online = 5 hours online gaming each week). For the second regression, predictors were average hours spent playing games each week in recent months: a) offline or b) online. For the third regression, predictors were average hours spent playing games each week in recent months: a) alone, b) with people they do not know, c) with people they only know online, and d) with people they know in real life. For each regression, all variables were entered as predictors on the same level. Mahalanobis distance was calculated to identify multivariate outliers in the associations between IGD score and our predictor variables (life stage regression = 3; online versus offline regression = 8; social regression = 8).

To correct for multiple comparisons, the Benjamini-Hochberg procedure was applied to correct for all group comparisons (n = 6) as well as the linear regression overall models and predictors (n = 13). All effects survived correction, unless otherwise stated.

#### Data availability.

We report how we determined our sample size and all data exclusions and manipulations. The raw data used in the analyses can be found on the Open Science Framework website here: https://doi.org/10.17605/OSF.IO/N36FE. The IGDS9 can be accessed through APA PsycTests; the LVUS is available upon request by emailing the corresponding author.

## Results

### GMM

**Fig 1:**
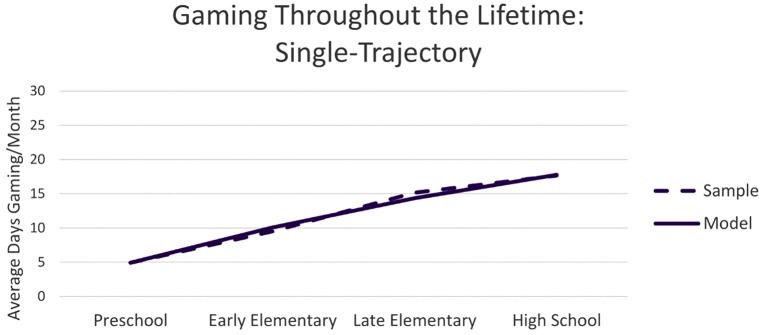
The model (solid line) and sample (dashed line) means for the single-trajectory model of days spent gaming each month during the first four life stages; preschool (ages 0–5), early elementary (ages 6–9), late elementary (ages 10–13), and high school (ages 14- 18).

The single-trajectory model found significant means and variance for the intercept (mean = 4.92, *p* < .001; variance = 66.7, *p* < .001), slope (mean = 5.60, *p* < .001; variance = 49.99, *p* < .001), and quadratic factor (mean = −0.44, p = .001; variance = 5.07, *p* < .001) suggesting multiple trajectories exist within the data and supporting the use of GMM (Fig 1). The analysis showed an acceptable fit to the data (χ^2^ difference [4] = 88.66 *p* < .001, CFI = .98, RMSEA = .13, SRMR = .03. It was decided to proceed with the GMM because the quadratic factor and variance were significant and all criteria showed sufficient fit besides the RMSEA, which does not perform well with small degrees of freedom [54].

**Fig 2:**
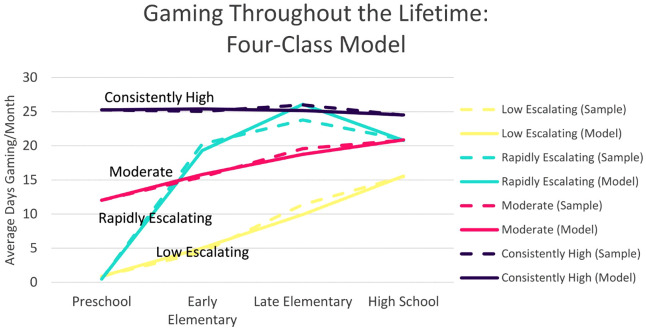
A visualization of the sample means (dashed line) and model means (solid line) of the four-class model of days spent gaming each month over the first four life stages: preschool (ages 0–5), early elementary (ages 6–9), late elementary (ages 10–13), and high school (ages 14–18). Low Escalating Gaming Group (Yellow) n = 334, Rapidly Escalating Gaming Group (Blue) n = 43, Moderate Gaming Group (Pink) n = 72, Consistently High Gaming Group (Purple) n = 51.

**Fig 3:**
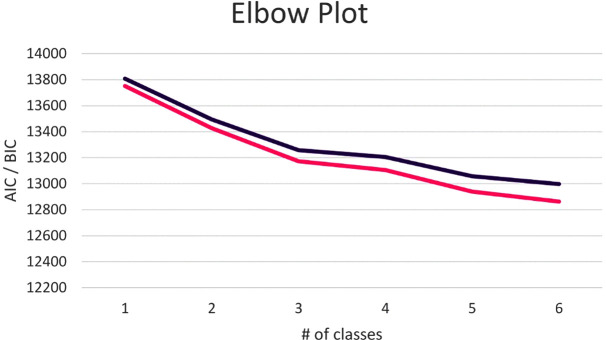
BIC (purple) and AIC (pink) values for 1-6 class models.

A series of GMM were conducted to determine the number of trajectories (one to six classes) that best fit the video game play data (Table 2). The likelihood ratio tests (VLMR LRT, LMR LRT, and BLRT) were significant for all models, entropy values were over 0.9 for all models, and the information criterion (AIC, BIC, and aBIC) decreased as classes were added to each successive model. We then considered the 4-, 5-, and 6-class models in terms of prevalence and interpretability [55]. The 5- and 6-class models both had two classes with similar trajectories characterized by high rates of gaming throughout the life stages. Both the 4-class model and the 6-class model had a group that reported very little gaming during preschool and high gaming at the rest of the life stages, accounting for ~8% of the sample. This group was not present in the 5-class model; each class in this model showed a similar, linear trajectory with different amounts of gaming during the preschool life stage. Since this group re-emerged in the 6-class model, it was considered a distinct class that would be lost if the 5-class model was adopted. These factors, in addition to advantages of parsimony, led to the selection of the 4-class model (shown in Fig 2 with growth parameters listed in Table 3). To provide a visual check on model fit, BIC and AIC values across the 1–6 class models were plotted. Although the largest improvements occurred up to the 3-class solution and no clear ‘elbow’ appeared at 4, the 4-class model captured a distinct trajectory, had high entropy (0.944), and adequate class sizes, supporting the robustness of this solution (Fig 3).

**Table 2:** Fit statistics for the 1- through 6-class model.

	1-class model	2-class model	3-class model	4-class model	5-class model	6-class model
Sample Size	500	77	51	43	21	21
		423	71	51	31	31
			378	72	53	39
				334	67	54
					328	67
						288
AIC	13751.24	13427.73	13173.12	13104.99	12940.07	12862.62
BIC	13807.82	13495.16	13257.47	13206.14	13058.08	12997.49
Adjusted BIC	13763.73	13444.37	13193.99	13129.96	12969.21	12895.92
Entropy	–	.96	.98	.95	.98	.96
VLMR LRT p-value	–	<.001	<.001	.015	.003	.009
LMR LRT	–	<.001	<.001	.017	.004	.01
BLRT	–	<.001	<.001	<.001	<.001	<.001

**Table 3:** Growth parameters for each class in the 4-class model.

*Group # (n)*	*Intercept (SE)*	*Linear Slope (SE)*	*Quadratic Slope (SE)*
1: Low Escalating Gaming (334)	0.88*** (.10)	3.79*** (.44)	0.37* (.16)
2: Rapidly Escalating Gaming (43)	0.48* (.24)	24.86*** (1.48)	−6.03*** (.51)
3: Moderate Gaming (72)	12.03*** (.40)	4.18*** (.96)	−0.411 (.31)
4: Consistently High Gaming (51)	25.27*** (.66)	0.32 (1.24)	−0.19 (.41)

* p < .05; ** p < .01; *** p < .001.

The majority of the sample belongs to Class 1 (66.8%, n = 334), which has a low intercept, a slow initial increase with a slight inverted U-shape between early elementary and high school; we refer to this as the “Low Escalating Gaming Group.” Class 2 (8.6%, n = 43) has a low intercept and a pronounced U-shape, with a significant increase in early elementary before decreasing slightly in high school; we refer to this as the “Rapidly Escalating Gaming Group.” Class 3 (14.4%, n = 72) has a moderate intercept and a slight increase over time; we refer to this as the “Moderate Gaming Group.” Class 4 (10.2%, n = 51) has a high intercept and remains steady over time; we refer to this as the “Consistently High Gaming Group.”

**Fig 4:**
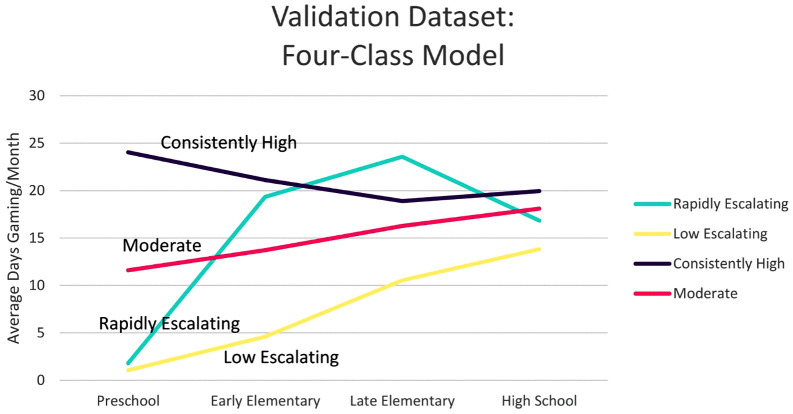
Model means of the 4-class solution in the independent validation sample (N = 202). Trajectory shapes and class proportions closely replicate those in the original sample: Low Escalating 65.0%, Moderate 15.6%, Consistently High 9.0%, Rapidly Escalating 10.4%.

The selected model was next applied to the independent validation sample (N = 202). A 2–6 class enumeration was conducted, model fit statistics in Table 4. The 4-class model preserved the trajectories identified in the original sample, including the Rapidly Escalating Group, and showed high entropy (0.944), indicating good class separation. Class proportions were consistent with the original sample, and trajectory shapes mirrored those in the original sample (Fig 4). However, the LMR and VLMR tests did not support the 4-class model over the 3-class model in this sample, although this is not unexpected given prior evidence demonstrating that likelihood ratio tests can show inflated rejection rates at smaller sample sizes [56]. Based on replication of class structure, interpretability, and consistency with the original sample, the 4-class model was retained in the validation dataset.

**Table 4:** Fit statistics for the 2- through 6-class model for the validation dataset.

	2-class model	3-class model	4-class model	5-class model	6-class model
Sample Size	28	18	18	11	3
	174	31	22	11	8
		153	31	23	9
			131	28	25
				129	30
					127
AIC	5283.858	5209.882	5163.672	5141.249	5131.639
BIC	5336.790	5276.048	5243.070	5233.881	5237.504
Adjusted BIC	5286.099	5212.684	5167.034	5145.171	5136.121
Entropy	.953	.968	.944	.947	0.981
VLMR LRT p-value	.0001	.0005	.4939	0.0200	.0370
LMR LRT	.0001	.0007	0.5054	0.0224	0.0388
BLRT	<.0001	<.0001	<.0001	<.0001	<.0001

The BCH procedure found a significant difference in IGD scores between the four gaming groups (*X*^*2*^ [3, N = 437] = 10.72, p = 0.01). Table 5 shows the IGD means and standard error for each group as well as the pairwise comparisons between groups. The Consistently High Group scored significantly higher than the Low Escalating Group and Rapidly Escalating Group. However, they did not score significantly higher than the moderate gaming group.

**Table 5:** A) IGD means and standard error for each gaming group; B) Comparisons of IGD scores among gaming groups.

A)	Group	Mean	SE
	Low Escalating	17.77	0.43
	Rapidly Escalating	16.58	1.31
	Moderate	19.20	1.10
	Consistently High	21.52	1.25
**B)**	**Group**	***X*** ^***2***^	***p*-value**
	Low Escalating vs. Rapidly Escalating	0.70	.40
	Low Escalating vs. Moderate	1.46	.23
	Low Escalating vs. Consistently High	8.12	.004 **
	Rapidly Escalating vs. Moderate	2.33	.13
	Rapidly Escalating vs. Consistently High	7.44	.006 **
	Moderate vs. Consistently High	1.93	.17

### Regression

#### Life stages.

The BCH procedure revealed the class with highest IGD score featured the highest levels of gaming in early childhood. To more formally test whether these earliest life stages could mark periods of susceptibility for later problematic gaming, we conducted a linear multiple regression modelling the relationship between IGD scores, days spent gaming at early life stages, and gender, which was significant (*F*[5, 430] = 5.59, *p* < .001, adjusted *R*^*2*^ = 0.05). Days spent gaming during preschool (β = 0.26, *p* < .001, semi-partial correlation [after controlling for the other life stages in the model] = 0.18) and high school (β = 0.15, *p* = .01, semi-partial correlation = 0.12) were identified as significant positive predictors of adulthood IGD scores; this indicated that more days spent gaming in preschool and high school is associated with higher current IGD scores in the present day. In contrast, days spent gaming during early elementary (β = −0.14, *p* = .08, semi-partial correlation = −0.08), late elementary (β = −0.06, *p* = .42, semi-partial correlation = −.04), and gender (β = −0.06, *p* = .25, semi-partial correlation = −.05) were unrelated to IGD scores.

#### Online versus offline gaming.

The regression modelling recent internet gaming behaviours (as hours spent gaming per week in recent months) indicated that the relationship between IGD scores, online versus offline gaming, and gender was significant (*F*[3, 432] = 14.68, *p* < .001, adjusted *R*^*2*^ = 0.06). Both time spent gaming online while controlling for offline gaming (β = 0.23, *p* < .001, semi-partial correlation = 0.22) and offline while controlling for online gaming (β = 0.11, *p* = .03, semi-partial correlation = .11) were found to be significant predictors of IGD scores. Gender was not found to be a significant predictor (β = −0.07, *p* = .20, semi-partial correlation = −.07). However, online gaming was more strongly predictive of IGD scores.

#### Social gaming.

The overall fit of the regression modelling of the relationship between IGD scores and the social aspects of gaming was significant (*F*[5, 430] = 6.91, *p* < .001, adjusted *R*^*2*^ = 0.06). All predictors involving time spent gaming with other people were significant while controlling for other social aspects of gaming, including: gaming with unknown people (β = 0.17, *p* < .001, semi-partial correlation = .17); gaming with people known only online (β = 0.13, p = .007, semi-partial correlation = .13); and gaming with people known in real life (β = 0.10, *p* = .04, semi-partial correlation = .10). Time spent gaming alone was not a significant predictor of IGD scores in the model (β = 0.06, *p* = .20, semi-partial correlation = .06) nor was gender (β = −0.06, *p* = .18, semi-partial correlation = −.06). This model suggests that the more hours spent gaming with others in recent months (regardless of your relationship to that person) is associated with higher IGD scores.

## Discussion

The current study examined whether the timing of frequent gaming during formative life stages predicted problematic gaming behaviours in adults. Using a GMM approach, we identified a 4-class model of video game engagement during childhood and adolescence that differentially predicted current IGD scores. As expected, the class with early onset high level gaming scored higher on IGD symptoms than the two classes which featured little to no early gaming. These results were supported by the regression analysis, which identified preschool as the life stage where video game engagement was most strongly associated with adult IGD symptoms, followed by gaming in high school. Conversely, time spent gaming in early and late elementary were not significant predictors of current IGD symptoms. Additionally, the current study aimed to identify social contexts in present day gaming that are associated with IGD symptoms. Although both were significant, online gaming was a stronger predictor of IGD symptoms than offline gaming. Contrary to predictions from the substance-related disorder research [40,41], gaming alone did not predict IGD symptom severity, yet all forms of social gaming were significant predictors. Overall, these findings in part mirror the substance-related disorder literature by highlighting childhood and adolescence as potential periods of heightened addiction susceptibility, though the two may differ when considering the exact ages of increased vulnerability.

### Clinical course

The observation that the earliest life stage (preschool) was most strongly associated with problematic gaming behaviours in adulthood does not appear to be driven by the sheer amount of total lifetime gaming. For example, the Rapidly Escalating Group showed similarly high levels of gaming as the Consistently High Group during the later life stages (late elementary and high school); yet, remarkably, they showed similarly low rates of adult problematic gaming behaviours as the Low Escalating group, which they resembled only in the low levels of preschool gaming. These results, paired with the life stage regression results, highlight that gaming during preschool (before age 6) is the best predictor of later life problematic gaming behaviours.

Although, this may indicate preschool years as the period of greatest susceptibility to adult problematic gaming, we cannot rule out the influence of unmeasured environmental factors in explaining our results, especially as environmental factors may account for up to 75% of variation in time spent gaming [57]. For example, higher rates of gaming at preschool ages may be associated with other caregiving factors (e.g., limited parental oversight) that predispose individuals towards both early gaming and the later IGD. Similar patterns are observed in gambling [23] and substance-related disorders [58,59], where early exposure is associated with poor family environments. It should be noted, however, that some early gaming exposure may currently reflect normative behaviour rather than a problematic childhood environment. Furthermore, animal models controlling for environmental variables support that early exposure impacts later outcomes for alcohol [60] and gambling disorders [61]. Future research should clarify how environmental risk factors relate to early video game engagement.

Though not as strong a predictor as preschool, high school was the only other life stage where video game engagement was found to significantly predict later problematic gaming. This is in line with past research that has identified adolescence as a period of addiction vulnerability [62]. Adolescence is characterized as a time of increased autonomy [63], but also reward seeking behaviour [64] against a backdrop of protracted development of inhibitory control [65,66]. This increased motivation towards rewards without developed inhibitory control mechanisms to moderate behaviours has been linked to addiction risk [67]. In our total sample, high school was the life stage with the highest average level of video game engagement. It is possible that frequent gaming during this period where inhibitory control is underdeveloped could lead to pathological reward learning which extends into adulthood. Interestingly, there is evidence that young adults with IGD display impaired reward learning and inhibitory control compared to healthy controls [68]. It is difficult to determine whether the reduction in inhibitory control is a predisposing versus resulting factor of problematic gaming, or some combination of the two; longitudinal research is needed to unravel directionality and potential interactions.

### How do contextual factors relate to problematic gaming behaviours?

This study identified two contextual factors associated with problematic gaming. As expected, online gaming was found to be a greater predictor of IGD symptoms than offline gaming [36,37], however, offline gaming was also a significant predictor. This supports the current conceptualization of “Gaming Disorder” in the *International Classification of Diseases*, which does not limit problematic gaming to only online gaming but rather includes specifiers for ‘predominately online’ or ‘predominately offline’ gaming [69]. The social context of the gaming was also significant; gaming with others, but not gaming alone, significantly predicted IGD symptoms. The strength with which gaming with others predicted IGD symptoms decreased with relationship closeness (unknown people > people known online > people known in real life). This pattern of association differs from that of substance-related disorders, where solitary use is associated with more severe addiction symptoms [40,41]. This may be because substance use and frequent gaming differentially impact social engagement. Substance-related disorders are strongly associated with family tension and interpersonal conflict [70]. In the IGD literature, online gaming frequency is negatively correlated with real life social support, but positively correlated with in-game social relationships [71]. One possibility is that problematic gamers may supplant real life relationships with online relationships. This is consistent with our finding that problematic gaming is most strongly related to gaming with strangers and least with real-life acquaintances. The risk factor may therefore not be online gaming per se, but lack of offline social support. Examining the motivations behind social and solitary gaming, and how these variables may evolve with addiction, may assist with the development of distinct treatment plans which target the underlying cause of problematic gaming.

### Limitations & future directions

While the results of this study found evidence that a relationship does exist between early gaming and later life problematic gaming, we are unable to draw definitive conclusions regarding the underlying mechanisms behind this relationship. For example, as in the substance use and gambling literature [1,72], early problematic gaming may be a coping mechanism for negative life events or a poor family environment [73,74]. Indeed, problematic gaming is associated with higher levels of externalizing and internalizing symptoms [75], and some have suggested that problematic gaming is an expression of a different underlying disorder [10]. Future research should carefully consider the potential influence of familial and social environment in assessing risk for NSRDs, and the timing of the emergence of problematic gaming relative to other mental health issues. It is also possible this relationship is driven by individual difference variables; previous research has found a positive relationship between IGD and impulsivity [76]. Impulsivity has also been related to early onset of substance use disorders [77] and gambling behaviours [78]. Identifying the underlying mechanisms by which early gaming and current problematic gaming behaviours are related in longitudinal experimental designs will be necessary for the development of preventative interventions for individuals at high risk for developing IGD.

In addition to these considerations, gender was not a significant predictor of IGD symptoms in any of our regression models. This finding is surprising given past research which consistently finds males are at a greater risk for problematic gaming [79]. One possibility is that gender differences in IGD risk are accounted for by differences in gaming behaviours, such as frequency, developmental timing, or online gaming preference, which were directly modelled in the present study. Future research should further examine whether gender has an independent effect on IGD risk or whether its influence is mediated through behavioural and contextual factors.

Another limitation is that this study used a cross-sectional retrospective design; retrospective measures may be subject to recall bias and/or an incorrect assessment of previous events or experiences [80]. In an attempt to mitigate the impact of recall bias, our instrument was modelled after validated retrospective lifetime substance use questionnaires, which have played a pivotal role in research and treatment [32,81] and included age ranges and schooling milestones as memory prompts. Recall bias seems less likely to explain our results given that the best predictors were distinct periods of development separated in time (i.e., not simple linear effect or additive effects). While the LVUS did have an acceptable test-retest reliability, longitudinal research which prospectively tracks video game usage and problematic gaming behaviours throughout childhood and adolescence is needed.

As our sample was recruited online, there is a chance that our participants were more comfortable with technology than the general population and, thus, may have been more likely to engage with video games. Using a recommended threshold of 32-points on the IGDS9-SF for identifying problematic gaming based on clinical studies [46,82], 7.2% of our sample met criteria, which is in line with existing prevalence estimates of IGD in adult populations [83]. Furthermore, their average weekly gaming in recent months (6.7 hours/ week) is also comparable to the global average (8.45 hours/week; [84]). Nevertheless, it may also be helpful for future research to be conducted with a clinical sample to conclusively identify gaming trajectories which predict clinical levels of IGD.

### Conclusion

The present study adds to the call for further research into IGD by examining factors that contribute to the condition’s development and severity. Using GMM, we identified a 4-class model of video game use during childhood and adolescence that differentially predicted adult IGD symptom severity. Notably, the class characterized by the highest level of preschool gaming exhibited significantly greater IGD symptoms than those with minimal early exposure, even when later gaming levels were similar. Across both GMM and regression analyses, gaming during early life (before age 6) best predicted problematic gaming symptoms in adulthood. This raises the possibility that an early onset of frequent gaming may have the greatest impact on later risk, paralleling the substance-related disorder literature. In addition, IGD symptoms were more strongly predicted by online gaming compared to offline gaming, in line with previous research. However, contrary to the substance use literature, gaming with others, but not alone, was positively associated with IGD scores, regardless of relationship closeness with their gaming partner. The current results may inform caregivers and paediatric guidelines regarding when and how video game play is introduced. Although it is tempting to conclude that consumers should avoid all early exposure to gaming, there are a number of unmeasured variables that remain to be explored. The present results should motivate further research to clarify how problematic gaming may emerge through interactions between developmental exposure, pre-existing mental health issues, home environmental factors, and individual traits.
